# Expression and Activity of Platelet Endothelial Nitric Oxide Synthase Are Decreased in Patients with Coronary Thrombosis and Stenosis

**Published:** 2019

**Authors:** Zahra Emami, Alireza Mesbah Namin, Javad Kojuri, Farideh Mashayekhi Jalali, Mozhgan Rasti

**Affiliations:** 1. Department of Biochemistry, Tarbiat Modaress University, Tehran, Iran; 2. Department of Biochemistry, Faculty of Medicine, Shiraz University of Medical Sciences, Shiraz, Iran; 3. Department of Cardiology, Faculty of Medicine, Shiraz University of Medical Sciences, Shiraz, Iran; 4. Department of Biochemistry, Faculty of Medicine, Arak University of Medical Sciences, Arak, Iran

**Keywords:** Cardiovascular diseases, eNOS, Nitric oxide, Platelets, Real time PCR, Thrombosis

## Abstract

**Background:**

Nitric Oxide (NO) which is synthesized by endothelial Nitric Oxide Synthase (eNOS) in both vascular tissues and platelets plays an important role as a protective mediator in the cardiovascular system. It modulates blood pressure, vasodilation and thrombosis. In this regard, eNOS activity and gene expression in platelets and NO levels in patients’ plasmas with Coronary Thrombosis (CT) and stenosis diseases were determined.

**Methods:**

Blood samples were collected from 60 subjects that where divided into three equal groups [without coronary artery disease, with CT and less than 70% Coronary Stenosis (CS)]. NO concentration in plasma was measured by the Griess reagent system. The eNOS activity was assessed based on a fluorimetric detection system in platelets and the gene expression was quantified by the real time-reverse transcription-polymerase chain reaction.

**Results:**

There was a significant decrease in the amount of NO concentration in the plasma of subjects with CT (0.53±0.09 *μM*, p<0.01) and CS (1.31±0.11 *μM*, p<0.01) compared to the control group (2.6±0.10 *μM*). The activity levels of eNOS enzyme were significantly lower in patients’ platelets with CT (0.68±0.013 *UF/mn*, p<0.01) and CS (0.85±0.017 *UF/mn*, *p*<0.01) than the control cases (1.29±0.019 *UF/mn*). These data were consistent with the reduction of the expression levels of eNOS in patients with CT (75 folds) and CS (4 folds) lower than the control cases.

**Conclusion:**

Patients with CT and CS possessed reduced eNOS activity and gene expression in their platelets. Decreased plasma concentration of NO in these patients confirmed the potential significance of the diagnostic and prognostic value of NO in the subjects’ plasma with vascular disease risk.

## Introduction

Nitric Oxide (NO) synthesized by endothelial nitric oxide synthase is an endothelium-derived relaxing factor and an important signaling messenger in the cardiovascular system 1. It regulates many biological functions such as vascular smooth muscle cell proliferation and migration, vascular tone, endothelial permeability, and the endothelial-leukocyte interaction, besides its antithrombotic effects 2.

NO is produced by the Nitric Oxide Synthase (NOS) family of enzymes from one of the chemically equivalent guanidino nitrogens of L-arginine. These enzymes need a number of essential cofactors such as calmodulin (CaM), tetrahydrobiopterin (H4B), flavin mononucleotide, FAD and NADPH in order to function 3. There are three distinct isoforms of NOS in mammalian cells including neuronal NOS (nNOS, Type I), inducible NOS (iNOS, Type II) and endothelial NOS (eNOS or Type III) 4.

eNOS generates NO in both vascular endothelial cells and platelets. The eNOS-generated NO plays a key role in the cardiovascular protective functions through inhibition of platelet aggregation, leukocyte endothelium adhesion, chemokine expression, inflammatory cell infiltration and reduction of vascular smooth muscle proliferation 5. The lack of these physiological effects of eNOS established hypertension and vascular abnormalities in eNOS knockout mice 1. In contrast, the synthesis of a high-level of NO by iNOS for long periods of time in response to agonists in vascular tissues is involved in pathophysiology of several diseases. Induction of iNOS expression in vascular tissues has been shown in endotoxic shock and cardiac allograft rejection 6,7. However, platelets do not have nuclei to induce iNOS transcription. Platelet agonists at the sites of vascular injury induce activation of the low-level of constitutively expressed iNOS along with eNOS to stimulate platelet activation and aggregation to form homeostatic thrombi 8.

The risk factor for the Coronary Artery Disease (CAD) is increased with endothelial dysfunction due to impairment in eNOS-generated NO as well as NO breakdown. Since interaction of blood platelets with arterial endothelium is important in the development of atherosclerosis and pathogenesis of CAD 9,10, thus eNOS is a potential candidate gene for studying atherosclerosis. The eNOS (NOS 3: chromosome 7q) is found at low levels in platelets but its product serves as an important deterrent to platelet-mediated arterial thrombosis 5. There is no data available in relation to increasing CAD risk and eNOS activity and gene expression levels in circular platelets *in vivo*. Both eNOS and iNOS enzymes are expressed in platelets. However, eNOS possesses the beneficial effects in the prevention of excesses activation and aggregation of platelets 5. So, this study was designed to analyze the activity and gene expression levels of eNOS in circulating platelets which are at a resting (not activated) state 11, from patients with endothelial dysfunction and thrombosis. In addition, the assessment of NO availability in plasma potentially plays an important role in the diagnostic and prognostic significance of subjects who are at risk for cardiovascular diseases 10. Therefore, NO concentrations were measured in patients’ plasmas with and without CAD. Our results confirmed the association between the decreased levels of NO in the plasma of patients with Coronary Thrombosis (CT) and stenosis diseases and for the first time, the reduced eNOS activity and gene expression in platelets of patients with CAD was shown.

## Materials and Methods

### Subjects and blood collection

All experiments with human subjects were approved by the ethic committee at Shiraz University of Medical Sciences. 60 subjects including patients with acute coronary syndrome signed a consent form for participating in this study. All subjects submitted to coronary angiography at Shiraz University of Medical Sciences Hospital were divided into three groups by the specialist. Among patients (60 cases), 20 cases did not have any CAD (control group), 20 patients were diagnosed with CT group, and the other 20 possessed less than 70% Coronary Stenosis (CS group). Venous blood was taken from subjects in sterile conditions with EDTA potassium salt as an anticoagulant. Platelets were isolated from patients’ blood by centrifuging (190×*g*) of whole blood for 15 *min*; and then centrifuging (2500×*g*) for 15 *min*. The pellet of platelet was resuspended and kept in Tyrode buffer (Sigma, USA) at 22°*C* until doing experiments. Platelets were counted using a Goryaev chamber. The plasma of samples was stored at −70°*C* for measuring NO concentration.

### NO concentration in plasma

In this experiment, NO concentration in plasma was measured by Griess reagent (Promega, USA) system. This system detects Nitrite (NO_2_^−^) which is one of the two primaries, stable and nonvolatile breakdown products of NO. The Griess Reagent System is based on the chemical reaction, which uses sulfanilamide and N-1-napthylethylenediamine dihydrochloride under acidic (phosphoric acid) condition. Measurement of NO concentration was performed in a 96-well microliter plate according to the manufacturer’s instructions. Absorbance was read after 10 *min* at 540 *nm* using a plate reader. Standards, controls and samples were measured in triplicate and expressed in *μM*.

### eNOS activity

The eNOS enzyme activity was assessed based on a fluorimetric detection system (BioTek, USA) in platelets and evaluated in Units of Fluorescence (UF) per *min* (m) per 10^6^ platelet cells (n) 12. Fluorescence of triazolofluoresceine, which is formed after interaction of NO with 4, 5-diaminofluoresceine formed from 4, 5-diaminofluoresceine diacetate under the action of intracellular esterase, was detected. The wavelengths of excitation and emission were 485 and 515 *nm*, respectively. Since endothelial NOS is only activated 13,14 in platelets, thus the activity of this particular isoform was only assayed and expressed as the *UF/mn*.

### RNA isolation, cDNA synthesis and real-time-reverse transcription-polymerase chain reaction (RT-PCR)

Total RNA was extracted from the platelet pellet using a RNAX-Plus kit (Cinnagen, Iran) and quantified by spectrophotometry at 260/280 *nm*. Reverse transcription was performed using a RevertAid ™ H Minus First Strand cDNA Synthesis Kit (Fermentas, Iran) in a total volume of 20 *μl*, using 1.5 *μg* of total RNA and random hexamer primers The relative copy numbers of eNOS and β-actin mRNAs in human platelets (as a target and an internal control) were determined by the sensitive method of real-time RT-PCR (Applied Biosystems Division, ABI 7500, v2.0.1). In each assay, both eNOS and β-actin mRNA copy numbers in platelets were measured in subjects with CT and CS in comparison to control group. The reaction mixture contained the PCR product 15 (1:15 diluted), each primer (5 *pM*), 1x of SYBR green DNA PCR Master Mix (Applied ABI Company, Foster City, CA USA) in a total volume of 20 *μl*. The following primers: eNOS: sense, 5′-CACCGCTACAACATCCTG-3′, antisense, 5′-GCCTT CTGCTCATTCTCC-3′; β-actin: sense, 5′-AGTCTTCCT TCCTGGGCAT-3′, antisense, 5′-CAGGAGGAGCAAT GATCT-3′ were used for amplification. Target primers were designed using primer express (Applied Biosystems) to span the region within two exons to avoid hybridization with genomic DNA sequences. Each sample was run in duplicate. Amplification was run for 10 *min* at 95°*C*, followed by 40 cycles of 15 *s* at 95°*C*, 70 *s* at 60°*C*. To check for the presence of non-specific PCR products or primer-dimers, a melting curve analysis was performed after the final PCR cycle. Efficiency of amplification was determined by a relative standard curve derived from fivefold serial dilutions of the PCR product. In all cases, the amplification efficiency was around 95%. The level of *eNOS* gene expression was normalized to the level of expression of the *β-actin* gene. 2^−ΔΔCT^ me-thod was used to calculate the relative expression level (fold changes) of *eNOS* gene in the platelets of patients’ samples. Data were analyzed by using the 7500 Software v2.0.1. Amplified fragments were separated in 1.5% agarose gel containing ethidium bromide to visualize and estimate the brightness of amplified fragments.

### Statistical analyses

Statistical analyses were carried out by SPSS software version 16. All the data were expressed as the mean±S.E.). Statistical differences were evaluated using the Student’s t-test. p<0.05 was taken to indicate a statistically significant difference. Kruskal-Wallis test (for continuous values) and Fisher’s exact test (for discrete variables) were used to compare the results of real time PCR across the three groups.

## Results

**Figure 1.**
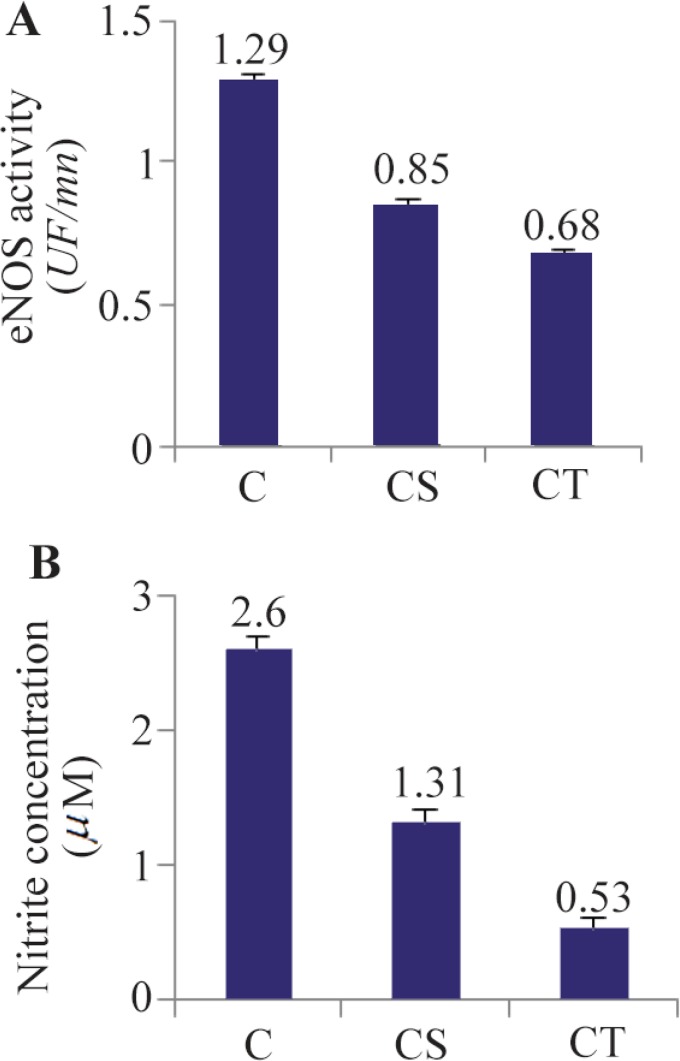
The eNOS enzyme activity of platelets and NO (Nitrite) levels in plasma of patients with arthrosclerosis. A) eNOS enzyme activity in platelets decreased significantly (2 tails, p<0.01) in Coronary Stenosis (CS) and Coronary Thrombosis (CT) patients compared to the control cases (C). B) NO levels in plasma were significantly lower in CS and CT groups (2 tails, p<0.01) than the control cases. All data were expressed as the mean±S.E.

The primary physiological function of platelets is forming homeostatic thrombi at the site of vascular injury 11. After exposure to immobilized adhesive proteins or soluble platelet agonists, stimulated iNOS and eNOS enzymes generate NO to induce platelet secretion and aggregation 8. Therefore, platelets in a normal circulation that were extracted from the subjects in this study were in a nonadherent (resting) state and were not activated 11. In this state, iNOS is not active so measuring iNOS activity in platelets was ignored. In addition, the role of eNOS is well established in endothelial dysfunction in knockout mice 1. Our results showed that the levels of eNOS enzyme activity in platelets were significantly lower in CS and CT groups (p<0.01) than in the control cases. The enzyme activity was decreased about 34% in the CS group and about 47% in the CT group compared to the control group (Figure 1A). Our findings demonstrated that CAD was consistent with reduced eNOS enzyme activity in platelets. It was previously shown that the main producer of NO in plasma is due to the constitutive activities of the vascular eNOS 16,17 and the plasma concentration of NO reflects the eNOS activity *in vivo* 10 and its dysfunction develops CAD 18. These findings were confirmed by analyzing NO levels in plasma of two groups with CAD in contrast to control cases. It was shown that there were significant reductions in the amounts of NO concentration in plasma by about 50 and 80% in CS and CT patients compared to the control cases, respectively (p<0.01, Figure 1 B).

**Figure 2.**
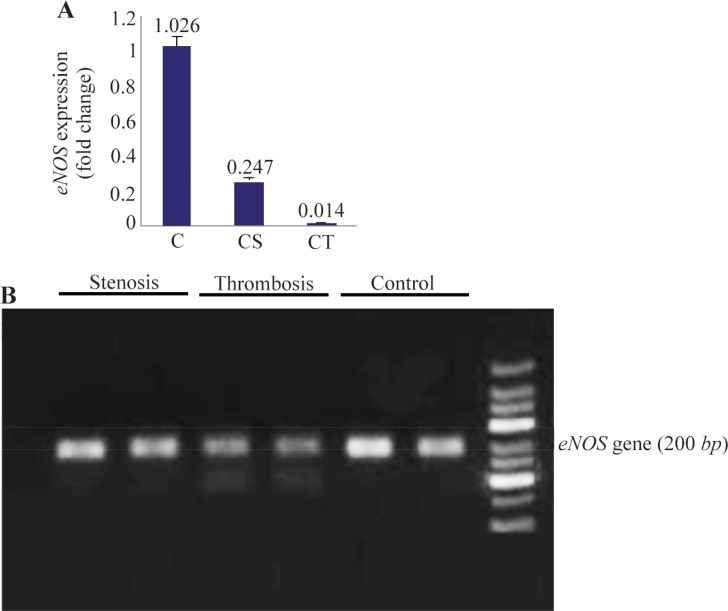
The eNOS relative expression levels in the platelets of patients’ samples and control group. A) eNOS mRNA decreased significantly (2 tails, p<0.01) in Coronary Stenosis (CS) and Coronary Thrombosis (CT) patients compared to the control cases (C). All data was expressed as the mean±S.E.. B) Gel electrophoresis of real-time PCR products of *eNOS* gene in three studied groups.

To study whether the reduction in NO release, by the eNOS enzyme in the platelets was caused by the impairment of its gene expression or not, real-time RT-PCR was conducted. Our data demonstrated that expression levels of eNOS mRNA (Figure 2A) were reduced significantly in CS group (4 folds, p<0.01) and CT group (75 folds, p<0.01) in comparison to the control cases. Figure 2B shows the amplified fragments from the three studied groups by real-time RT-PCR. Further, the correlation between the NO concentration in plasma, the levels of eNOS activity and its mRNA expression in platelets of all studied groups was examined. Regression analysis of these data for both CT and CS patients showed that there was a correlation between the low level of NO concentration in patients’ plasmas and the decreased levels of activity and expression of eNOS in their platelets (2 tails, p<0.01). It was assumed that the reduction of plasma concentration of NO was the result of decreased vascular eNOS enzyme activity in these patients. In this regard, it is suggested that the same pathological reason may be involved in the reduction of eNOS activity in vascular and platelets of patients with CS and CT diseases.

## Discussion

The role of platelet-derived NO in physiological homeostasis and pathologic thrombosis that complicates the course of CAD has been controversial 6,8,17. Platelet-derived NO is an important modulator of platelet functions including adhesion and aggregation. However, platelets produce much less NO by eNOS enzyme than do endothelial cells. eNOS-generated NO in platelet is important in situations where platelets are close to each other, and specifically in the region of the growing thrombus 19. It has been shown when platelet NO release is diminished, it is accompanied by an increase in pathogenesis of thrombotic complication of vascular disorders 20. There is evidence that the platelet NO production is impaired in patients with CAD and there is an inverse correlation between NO production in platelet and the number of coronary risk factors such as age, smoking and mean arterial pressure 21. The low level of iNOS enzyme is constitutively expressed in platelets. It can be only activated when exposed to the platelet agonists such as thrombin, collagen or the other agonists at the sites of vascular injury during platelet activation. It has been shown that iNOS knock down or using iNOS inhibitors in platelets decreases agonist-induced platelet secretion and aggregation. Moreover, the iNOS knockout mice showed prolonged bleeding time. These findings confirm that the activation of iNOS occurs in activated platelets in order to form homeostatic thrombi 8. Therefore, iNOS was not activated in the circulating platelets that were analyzed in this study.

The results of the present study provide evidence that arthrosclerosis is accompanied with reduced eNOS activity and gene expression in patients’ platelets with CT and CS. It was shown that the reduction of the eNOS enzyme activity of platelets in these patients is because of its low eNOS mRNA expression. As a result, their platelets produce low levels of NO which contributes to decreased concentration of NO in patients’ plasmas. Similar results were obtained in patients with major depressive disorder who showed dramatically lower platelet eNOS activity and plasma level of NO compared with healthy controls. This reduced NO production, by platelets and the endothelium was hypothesized as the main reason for the increased risk of developing cardiovascular disease in these patients 22. In addition, another study showed smokers who had low platelet NO synthesis which resulted in thrombus formation and developed into acute coronary syndromes. It was found that cigarette smoking and diabetes mellitus were significantly negative predictors and antioxidant (vitamin E) treatment was a significantly positive predictor of platelet eNOS mRNA expression 5. eNOS-generated NO by endothelial cells and platelet prevents platelet adhesion to vascular wall and platelet aggregation. It plays a key role in the inhibition of thrombus formation 20.

On the other hand, most of the studies have been done on the level of eNOS expression in vessels 23. It has been reported that both eNOS expression and activity were significantly reduced in right atrial tissue from patients with CAD 18. Other studies have been shown that levels of eNOS mRNA are increased by shear stress 4,24,25, estrogen 26 and exercise 27, but these levels are decreased during heart failure and possibly other disease states in vessels 23. It is consistent with our study that the level of eNOS expression in platelets was also decreased during CT and CS. In addition, it correlates with decreased levels of NO plasma concentrations in these patients. Plasma concentrations of NO have been used as a marker of vascular NO generation 10,16,17; therefore, reduced plasma concentration of NO in our patients with CAD which represents low eNOS activity in their vascular vessels is consistent with previous results. The pathogenesis of atherosclerosis is based on the interaction of blood elements such as platelets and leukocytes as well as lipids with the arterial wall. It has been shown that the plaque rupture leads to the exposure of collagen and vessel media that activate platelets and the coagulation cascade to make occlusive thrombus formation. It leads to myocardial ischemia that can ultimately result in myocardial cell injury and necrosis 20. However, how artery diseases affect platelets eNOS expression or vice versa and its molecular mechanisms should be further elucidated.

In this study, it was shown that the levels of NO plasma concentration in patients with CT and CS were significantly lower than the control cases. It has been shown that 70±5% of plasma nitrite is derived from constitutive vascular eNOS activity 16. The iNOS transcription is induced in response to inflammatory mediators such as cytokines, lipopolysaccharides and others in vascular tissues. For instance, over production of NO is the main cause of endotoxic shock 6. Most NO in non-inflammatory conditions comes from the eNOS endothelium and it is an important atheroprotective mediator. The defect in NO biological activity, which is because of either a decrease in its synthesis and conservation or accelerated breakdown due to oxidative stress, has been associated with increases in cardiovascular risk factors 28,29. Regarding the important effects of NO deficiency on the increase of cardiovascular disease risk, vascular eNOS activity as an index of NO bioactivity can be assessed by measuring plasma level of nitrite^17^. Our results also showed this is of diagnostic and prognostic value in patients with CT and CS.

## Conclusion

Our study showed that eNOS activity and expression decreased in patients’ platelets with CT and CS. Therefore, NO generation is reduced in platelets that are accompanied with the low level of NO plasma concentration in these patients. However, the molecular mechanisms that have effect on eNOS mRNA expression in cardiovascular disease should be studied in the future.
